# Association between albumin or prealbumin levels at different stages and prognosis in severe acute pancreatitis: a 5-year retrospective study

**DOI:** 10.1038/s41598-022-21278-1

**Published:** 2022-10-06

**Authors:** Tongtian Ni, Yi Wen, Yihui Wang, Weisong Jiang, Huiqiu Sheng, Erzhen Chen, Enqiang Mao, Zhixin Lan, Yaguo Huang, Yuhua Zhou

**Affiliations:** 1 Department of Emergency, Ruijin Hospital, Shanghai Jiao Tong University School of Medicine, 197 Ruijin Er Road, Huangpu District, Shanghai, 200025 China; 2 Department of Critical Care and Emergency, Wuxi Branch of Ruijin Hospital, 197 Zhixian Road, Xinwu District, Wuxi, 214000 China

**Keywords:** Acute pancreatitis, Outcomes research

## Abstract

Severe acute pancreatitis (SAP) is a common abdominal disorder contributing to high mortality and open laparotomy rates. The role of exogenous infused albumin in fluid resuscitation or continuous therapy has always been an unanswered question. In early stage after onset, SAP patients with higher serum albumin or prealbumin show a better prognosis. In this study, we tried to disclose the linkage between albumin/prealbumin and SAP prognosis and establish a new goal-directed therapy involving albumin and prealbumin. Pearson’s chi-squared test and the Mann–Whitney U test were used to compare the descriptive data between surviving and non-surviving patients. Three days, 4–7 days, 8–14 days and 15–28 days after SAP onset were defined as stages 1–4. The average concentrations of albumin and prealbumin were calculated, and receiver operating characteristic (ROC) curves were drawn to screen out the best cutoff values associated with poor prognostic outcomes, including laparotomy and failure to survive. Kaplan–Meier survival curves and log-rank tests were used to validate the effect of the cut-off values. A total of 199 admitted patients were enrolled in this study. According to the analysis of the ROC curve, the serum albumin value should be raised to 35, 37, 40 and 42 g/L in the 1–4 stage after onset. Serum prealbumin values should be raised to 108, 180, and 181 g/L in stages 2–4 after onset. The validity of the above data was confirmed by Kaplan–Meier survival curves. Serum albumin and prealbumin levels in the early stage of SAP are significantly relevant to prognosis. Albumin should be infused from the fluid resuscitation stage to continuous therapy in order to reach the targets mentioned above. The increase in prealbumin depends on the early initiation of enteral nutrition and this also helps to ameliorate the prognosis.

## Introduction

Severe acute pancreatitis (SAP) is a common abdominal disorder. As a subtype of acute pancreatitis associated with multiple organ failure and systemic inflammatory response syndrome, it is a main reason for ICU admission, which results in a long hospitalization time and high medical expenses^1,2^. The prognosis of SAP varies according to the timely implementation of early-stage fluid resuscitation, appropriate antibiotic usage, nutritional support, intervention measures aimed at aetiology, etc. With the progress in nonsurgical treatment of SAP, laparotomy rates and mortality are important criteria for evaluating prognosis and affect the quality of life of patients^3,4^.

The intensive management of SAP in our hospital, which includes a bundle of treatments, has proven its efficacy in the last 10 years of clinical practice ^4,5^. In addition to the mentioned measures in such treatment, we noticed that elevation of serum albumin concentration levels in acute and subacute stages of SAP obviously affects the prognosis. According to our clinical experience, patients with relatively higher serum albumin levels or quicker recovery of serum prealbumin levels in the first 28 days, especially in the first 14 days after the onset of disorder, often have better prognosis than patients with persistent low-level albumin and unrecoverable prealbumin levels.

The predictive albumin-related values have been proven to be related to SAP prognosis. Similarly, higher C-reactive protein-albumin or creatinine-albumin ratios are linked to worse clinical outcomes, including a higher Ranson score, Atlanta classification and rate of surgical debridement and in-hospital mortality^6,7^. All these previous studies suggest that albumin is an important factor in the process of SAP treatment and is worth considering. In our clinical practice, we found that the serum albumin concentration could independently predict poor prognosis instead of combined parameters such as C-reactive protein-albumin or creatinine-albumin ratios. Therefore, in this study, the average serum albumin concentration value was recorded, and its predictive values for consequent laparotomy and in-hospital mortality were disclosed. Moreover, a rapid recovery of serum prealbumin levels was also found to be a predictive factor for good clinical results.

## Results

After being screened from the raw data, 199 patients were formally enrolled in this study. Table 1 shows the patients’ descriptive data. In terms of survival, patients were divided into two groups, and a series of variables were compared by using the Mann–Whitney U test and the Pearson Chi-square test. Regarding general data, the average age of the nonsurvival group was 52.33 years, and that of the survival group was 45.57 years, with the nonsurvival group being significantly older than the survival group (P = 0.012). The ratio of males to females and the BMI showed no significant difference (P = 0.837 and 0.204). Moreover, nonsurviving patients had a lower average SBP (P = 0.015). With respect to aetiology, the nonsurvival group contained more biliary-origin patients, and hypertriglyceridaemia-origin patients were more likely to be in the survival group (P = 0.017 and 0.038). Among the laboratory examination indicators sampled at the very beginning of admission, nonsurviving patients showed higher procalcitonin (PCT), serum amylase (AMY), total bilirubin (TBIL), blood urea nitrogen (BUN), and serum creatinine (SCR) levels and lower albumin levels (P = 0.002, 0.022, < 0.001, < 0.001 and 0.003). Regarding the critical care scores, all modified Marshall, BISAP and APACHE II scores showed significant differences between the two groups (P < 0.001, 0.002 and < 0.001).

**Table 1:** Characteristics of patients with severe acute pancreatitis on admission (n%; $\overline{x }$±s).

	Nonsurvival (n = 33)	Survival (n = 166)	*P* value
Mean age (years)	52.33.09 ± 15.01	45.57 ± 13.76	0.012*
Gender (male%)	24 (72.73%)	116 (69.88%)	0.837
Body Mass Index (kg/m^2^)	24.59 ± 5.08	25.64 ± 4.18	0.204
**Aetiology**			
Biliary	23 (69.70%)	78 (46.99%)	0.017*
Hypertriglyceridaemia	7 (21.21%)	67 (40.36%)	0.038*
Alcohol	2 (6.06%)	8 (4.82%)	0.766
Others	1 (3.03%)	13 (7.83%)	0.325
**Comorbidities**			
Hypertension (%)	10 (30.30%)	56 (33.73%)	0.702
Diabetes mellitus (%)	8 (24.24%)	40 (24.10%)	0.986
**Indicators**			
White blood cell (× 10^9^/l)	14.17 ± 6.41	13.61 ± 5.39	0.598
C-reactive protein (µg/ml)	129.14 ± 81.36	151.46 ± 112.66	0.280
Procalcitonin (ng/ml)	11.69 ± 16.17	5.06 ± 9.59	0.002*
Serum amylase (U/L)	1347.61 ± 895.61	939.32 ± 938.14	0.022*
Alanine aminotransferase (U/l)	64.39 ± 157.69	46.20 ± 72.45	0.300
Total bilirubin (µmol/l)	48.30 ± 53.21	31.53 ± 40.27	0.040*
Blood urea nitrogen (mmol/l)	15.53 ± 10.82	7.00 ± 6.16	< 0.001*
Serum creatinine (µmol/l)	253.70 ± 257.08	101.11 ± 148.41	< 0.001*
Albumin (g/L)	31.12 ± 6.15	34.93 ± 6.72	0.003*
Prealbumin (g/L)	123.39 ± 83.59	154.88 ± 67.33	0.079
Oxygenation index	189.20 ± 69.84	199.76 ± 70.75	0.434
SBP (mmHg)	121.24 ± 25.21	133.96 ± 27.69	0.015*
Modified Marshall score	4.45 ± 1.68	2.87 ± 1.17	< 0.001*
BISAP score	2.91 ± 0.68	2.51 ± 0.66	0.002*
APACHE II score	14.70 ± 5.43	10.58 ± 4.61	< 0.001*
Duration of ICU stay (days)	47.85 ± 55.82	32.32 ± 33.88	0.131

*SBP* systolic blood pressure, *BISAP score* Bedside Index for Severity in Acute Pancreatitis score, *APACHE II score* Acute Physiology and Chronic Health Evaluation II score.

*P < 0.05 was considered statistically significant.

The study-defined stage average albumin/prealbumin levels of the surviving and nonsurviving patients were listed in Table 2. Except for the average prealbumin level in stage 1 (P = 0.066), all albumin/prealbumin levels were significantly lower than those of the survival group. In Table 3, patients are redivided by the presence of laparotomy. Except for the average prealbumin level in stages 1 and 2 (P = 0.766 and 0.052), the albumin/prealbumin levels were also significantly lower.

**Table 2:** Comparison of albumin and prealbumin in different stages of SAP patients in survival group and non-survival group.

		Non-survival	Survival	P
**Average serum albumin concentration (g/L)**				
Stage 1		n = 33	n = 166	
	Number of detections	3 (0)	3 (1)	0.603
	Value	33.16 ± 5.61	35.71 ± 5.00	0.012
Stage 2		n = 31	n = 166	
	Number of detections	4 (0)	3 (2)	< 0.001
	Value	37.08 ± 6.13	40.78 ± 5.44	0.004
Stage 3		n = 29	n = 166	
	Number of detections	5 (4)	2 (3)	< 0.001
	Value	37.80 ± 6.72	43.02 ± 6.06	< 0.001
Stage 4		n = 23	n = 154	
	Number of detections	8 (8)	4 (3)	< 0.001
	Value	35.21 ± 4.82	42.91 ± 5.95	< 0.001
**Average serum prealbumin concentration (g/L)**				
Stage 1		n = 33	n = 166	
	Number of detections	3 (2)	2 (1)	0.636
	Value	113.97 ± 56.56	131.59 ± 49.38	0.066
Stage 2		n = 31	n = 166	
	Number of detections	4 (0)	3 (2)	0.002
	Value	105.82 ± 39.41	125.44 ± 43.50	0.024
Stage 3		n = 29	n = 166	
	Number of detections	5 (4)	2 (3)	< 0.001
	Value	123.14 ± 42.45	197.79 ± 78.21	< 0.001
Stage 4		n = 23	n = 153	
	Number of detections	8 (9)	4 (2)	< 0.001
	Value	132.06 ± 65.01	243.40 ± 77.78	< 0.001

Data are presented as medians (interquartile range) or mean ± standard deviation. P values were calculated using nonparametric test.

**Table 3:** Comparison of average serum albumin/prealbumin concentrations between laparotomy and nonlaparotomy patients.

		Laparotomy	Non-laparotomy	P
**Average serum albumin concentration (g/L)**				
Stage 1		n = 34	n = 165	
	Number of detections	3 (0.25)	3 (0.5)	0.946
	Value	33.83 ± 5.21	35.58 ± 5.14	0.038
Stage 2		n = 34	n = 163	
	Number of detections	4 (1.25)	3 (2)	0.120
	Value	37.02 ± 5.79	40.86 ± 5.47	< 0.001
Stage 3		n = 34	n = 161	
	Number of detections	6 (5)	3 (2)	< 0.001
	Value	38.52 ± 5.95	43.03 ± 6.25	< 0.001
Stage 4		n = 34	n = 143	
	Number of detections	8.5 (9.25)	4 (2)	< 0.001
	Value	37.94 ± 6.59	42.85 ± 5.95	< 0.001
**Average serum prealbumin concentration (g/L)**				
Stage 1		n = 34	n = 165	
	Number of detections	3 (1)	2 (1)	0.593
	Value	130.80 ± 56.45	128.23 ± 49.87	0.766
Stage 2		n = 34	n = 163	
	Number of detections	4 (1.25)	3 (2)	0.145
	Value	108.66 ± 44.18	125.20 ± 42.80	0.052
Stage 3		n = 34	n = 161	
	Number of detections	6 (5)	3 (2)	< 0.001
	Value	124.80 ± 58.30	199.76 ± 76.14	< 0.001
Stage 4		n = 34	n = 142	
	Number of detections	8.5 (10)	4 (2)	< 0.001
	Value	134.55 ± 54.35	251.43 ± 74.77	< 0.001

Number of detections means the median and interquartile times of albumin/prealbumin detections in the corresponding stage of every patient.

**Figure 1:**
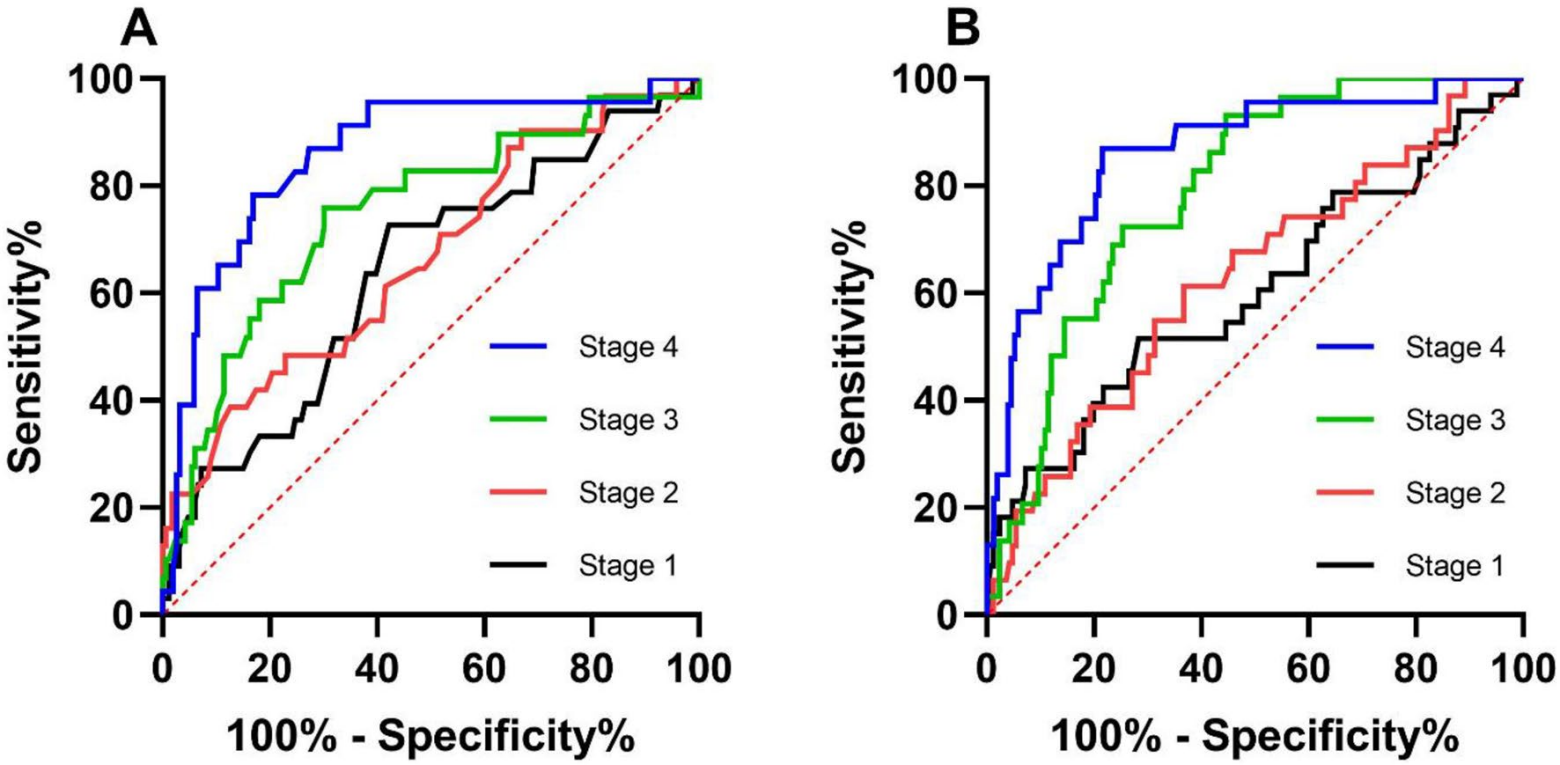
ROC curve of the albumin/prealbumin concentration for survival in SAP. (**A**) The ROC curve of albumin, and (**B**) the ROC curve of prealbumin.

**Figure 2:**
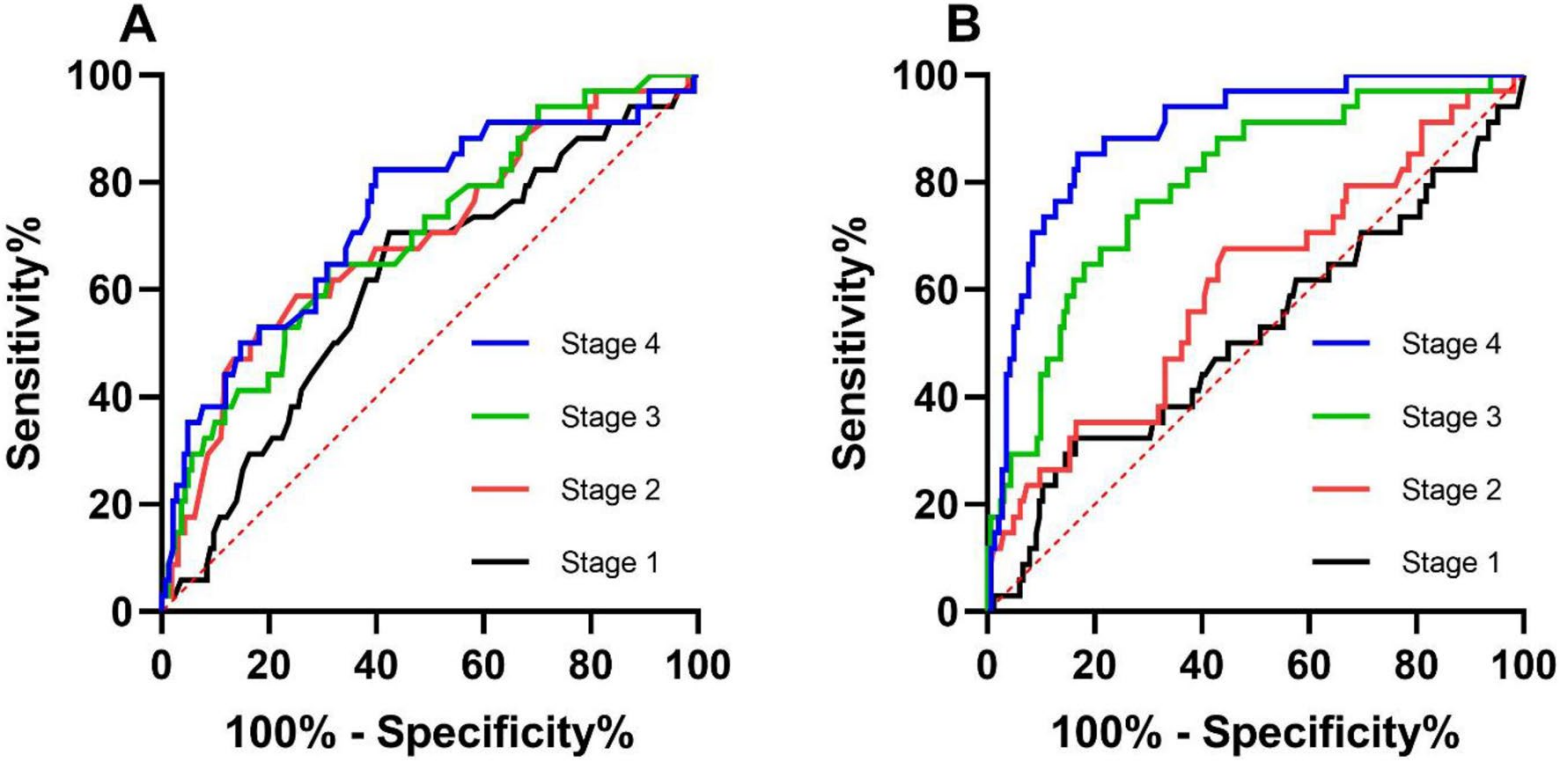
ROC curve of the albumin/prealbumin concentration for laparotomy in SAP. (**A**) The ROC curve of albumin, and (**B**) the ROC curve of prealbumin.

The relationships between albumin/prealbumin level and prognosis, including survival or nonlaparotomy, were described by ROC and cut-off values of different stages. In the ROC of albumin predicting survival, the AUCs from stage 1 to 4 were 0.638, 0.664, 0.737 and 0.860 (P < 0.05). Correspondingly, the cut-off values were 34.83 g/L, 34.13 g/L, 39.88 g/L and 37.47 g/L. In the ROC of prealbumin, the curve of stage 1 lacked predictive ability because P = 0.066. The AUCs from stages 2 to 4 were 0.627, 0.790 and 0.861, respectively (P < 0.05). The cut-off values were 107.6 g/L, 179.5 g/L and 180.7 g/L (Table 4; Fig. 1). In the ROC of albumin predicting the nonlaparotomy rate, the AUCs from stage 1 to 4 were 0.613, 0.693, 0.698 and 0.737 (P < 0.05) with cut-off values of 34.83 g/L, 36.13 g/L, 39.88 g/L and 41.38 g/L. In the ROCs of prealbumin, the curves of stages 1 and 2 were meaningless (P = 0.765 and 0.052). The AUCs of stages 3 and 4 were 0.794 and 0.893, respectively, with cut-off values of 145.6 g/L and 180.7 g/L, respectively (Table 5; Fig. 2).

**Table 4:** ROC curve of albumin and prealbumin for the death of patients with severe acute pancreatitis.

Indicators	Stage	AUC	95% CI	Cut-off	Sensitivity	Specificity	P value
Serum albumin (g/L)	1	0.638	0.532–0.744	34.83	0.727	0.578	0.013*
	2	0.664	0.556–0.772	34.13	0.387	0.874	0.004*
	3	0.747	0.644–0.850	39.88	0.759	0.699	< 0.001*
	4	0.860	0.775–0.945	37.47	0.783	0.831	< 0.001*
Serum prealbumin (g/L)	1	0.601	0.486–0.717	104.8	0.515	0.717	0.066
	2	0.627	0.518–0.736	107.6	0.613	0.633	0.025*
	3	0.790	0.715–0.864	179.5	0.931	0.554	< 0.001*
	4	0.861	0.778–0.945	180.7	0.870	0.784	< 0.001*

**Table 5:** ROC curve of albumin and prealbumin for the laparotomy of patients with severe acute pancreatitis.

Indicators	Stage	AUC	95% CI	Cut-off	Sensitivity	Specificity	P value
Serum albumin (g/L)	1	0.613	0.510–0.717	34.83	0.706	0.576	0.038*
	2	0.693	0.591–0.795	36.13	0.529	0.816	< 0.001*
	3	0.698	0.601–0.796	39.88	0.647	0.689	< 0.001*
	4	0.737	0.638–0.835	41.38	0.824	0.601	< 0.001*
Serum prealbumin (g/L)	1	0.516	0.400–0.633	172.5	0.324	0.836	0.765
	2	0.606	0.497–0.715	118.6	0.677	0.558	0.052
	3	0.794	0.712–0.877	145.6	0.765	0.721	< 0.001*
	4	0.893	0.836–0.949	180.7	0.853	0.831	< 0.001*

**Figure 3:**
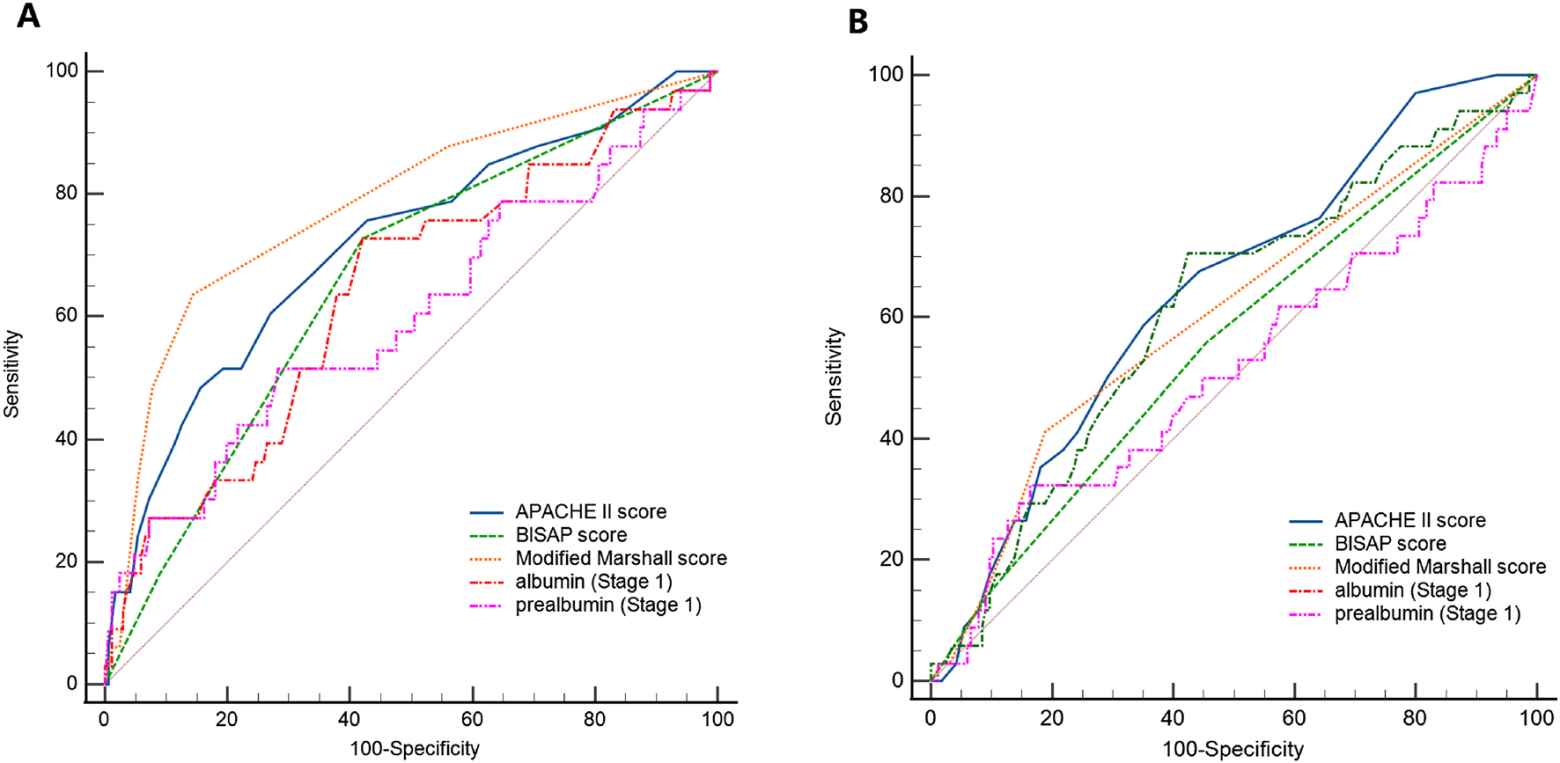
ROC curves of the APACHE II score, BISAP score, Modified Marshall score, first stage albumin and prealbumin for prognosis. (**A**) The comparison of ROC curves for nonsurvival. (**B**) The comparison of ROC curves for laparotomy.

Although the serum albumin/prealbumin was the main aim of this study, the traditional scoring systems were also classical method to predict the prognosis. The relationship between scoring systems (APACHE II score, BISAP score, and modified Marshall score) and prognosis was also shown by ROC curves. We found that the Marshall score had the highest value in predicting mortality in the first stage. It was statistically significantly different from the predictive value of albumin and prealbumin. But the value of albumin and prealbumin in predicting mortality began to increase as the disease progressed. By the fourth stage, their value in predicting mortality has been significantly higher than that of APACHE II score and BISAP score. Although the AUC value was higher than the Marshall score, it was not statistically significant (Table 6; Fig. 3). In terms of laparotomy rate, traditional scores were not of high predictive value, which may be due to some subjective factors in judging the implementation of laparotomy. But even so, prealbumin was significantly more effective at predicting laparotomy rate than these three scores at stage 3 and stage 4 (Table 7; Fig. 3).

**Table 6:** Comparison of ROC curves for survival in SAP.

Indicators	Stage	AUC	95% CI
Serum albumin (g/L)	1	0.638	0.532–0.744*
	2	0.664	0.556–0.772
	3	0.747	0.644–0.850
	4	0.860	0.775–0.945^#^∇
Serum prealbumin (g/L)	1	0.601	0.486–0.717*
	2	0.627	0.518–0.736
	3	0.790	0.715–0.864∇
	4	0.861	0.778–0.945^#^∇
Modified Marshall score	On admission	0.782	0.718–0.837
APACHE II score	On admission	0.715	0.647–0.776
BISAP score	On admission	0.658	0.588–0.724*

*P < 0.05 compared with Modified Marshall score. ^#^P < 0.05 compared with APACHE II score. ∇P < 0.05 compared with BISAP score.

**Table 7:** Comparison of ROC curves for laparotomy in SAP.

Indicators	Stage	AUC	95% CI
Serum albumin (g/L)	1	0.613	0.510–0.717
	2	0.693	0.591–0.795
	3	0.698	0.601–0.796
	4	0.737	0.638–0.835∇
Serum prealbumin (g/L)	1	0.516	0.400–0.633
	2	0.606	0.497–0.715
	3	0.794	0.712–0.877*^#^∇
	4	0.893	0.836–0.949*^#^∇
Modified Marshall score	On admission	0.606	0.543–0.674
APACHE II score	On admission	0.642	0.572–0.709
BISAP score	On admission	0.558	0.487–0.629

*P < 0.05 compared with Modified Marshall score. ^#^*P* < 0.05 compared with APACHE II score. ∇*P* < 0.05 compared with BISAP score.

**Figure 4:**
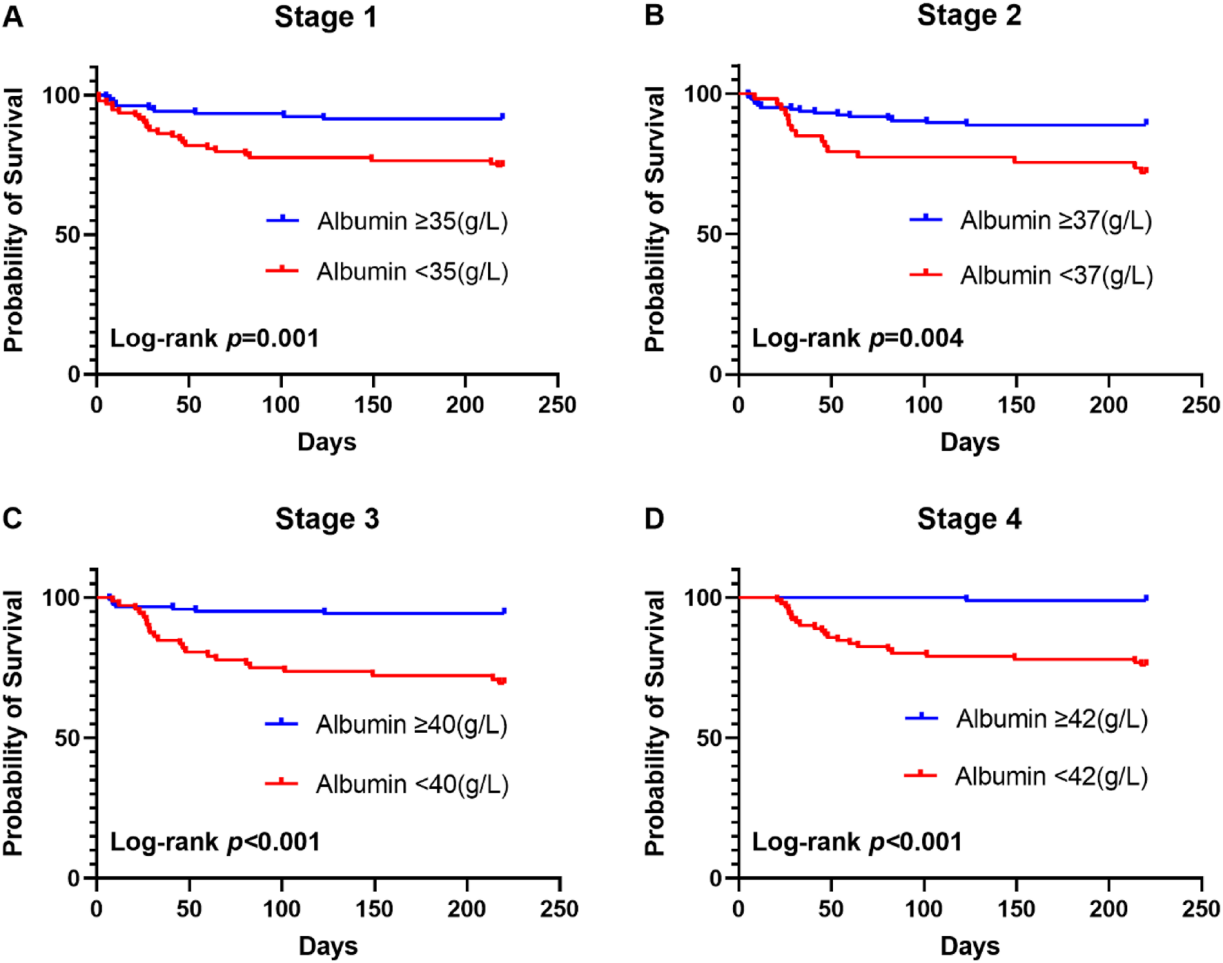
Kaplan–Meier 240-day survival between albumin target and non-target groups at different stages in patients with severe acute pancreatitis.

**Figure 5:**
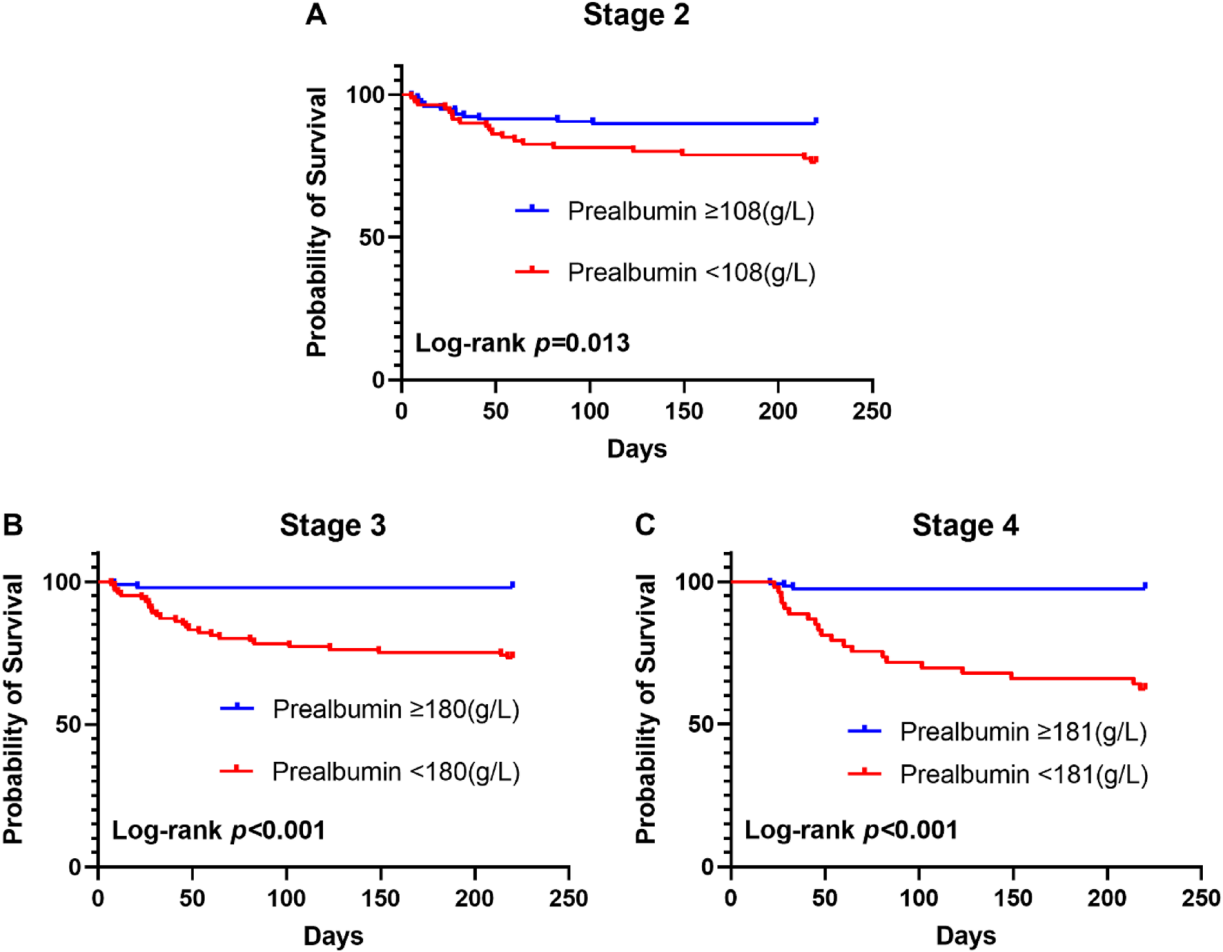
Kaplan–Meier 240-day survival between prealbumin target and non-target groups at different stages in patients with severe acute pancreatitis.

The effect of cut-off value was validated by Kaplan–Meier survival curves and log-rank tests. From stage 1 to 4, patients with albumin levels reaching or exceeding cut-off values possessed higher survival probability and the Log-rank P value were 0.001, 0.004, < 0.001 and < 0.001 (Fig. 4). From stage 2 to 4, patients with cut-off value defined prealbumin levels were also with higher survival probability and the Log-rank p value were 0.013, < 0.001 and < 0.001 (Fig. 5).

## Discussion

Nearly 20–30% of patients experiencing acute pancreatitis develop a severe form that is associated with single or multiple organ dysfunction and thus causes high medical expenses and worse prognosis^1,5^. According to the intensive management proposed by Mao et al., controlled fluid resuscitation and nutrition support are two emphasized aspects in the process of SAP early-stage treatment^5^. Although fluid resuscitation is performed for patients exhibiting sepsis or hypovolemia, whether colloid is superior to crystal in ameliorating short-term mortality is still controversial^8,9^. Natural colloids, such as albumin, may still have beneficial effects in preserving organ functions and relieving capillary leakage^10^. Early goal-directed therapies in the field of fluid resuscitation have been widely discussed. Whether patients benefit from it or not, goal-directed therapy helps clinicians provide more targeted treatments.

In our clinical practice and according to SAP treatment experiences, it is found that patients with higher levels of serum albumin or prealbumin often have a better prognosis, which means avoiding opening laparotomy in the following stage and restoring quality of life after clinical intervention. Regardless of exogenous supplementation with albumin or the presence of a high level at the onset time point of SAP, efficient optimization of serum albumin or prealbumin was significantly relevant to the clinical results. However, merely relying on clinical experience always leads to a lack of reference and makes blind treatment, which may cause the prognosis to be unpredictable.

Goal-directed therapies have been widely endorsed in different fields of clinical treatment, especially in fluid resuscitation in the early stage of septic shock^11–13^. Translationally using the theory of goal-directed therapy in fluid treatment and based on the clinical reality in our experience, we tried to set the goal of serum albumin/prealbumin levels in the different stages of SAP and to make the strategy clearer when fluid resuscitation and nutrition support are being provided.

**Figure 6:**
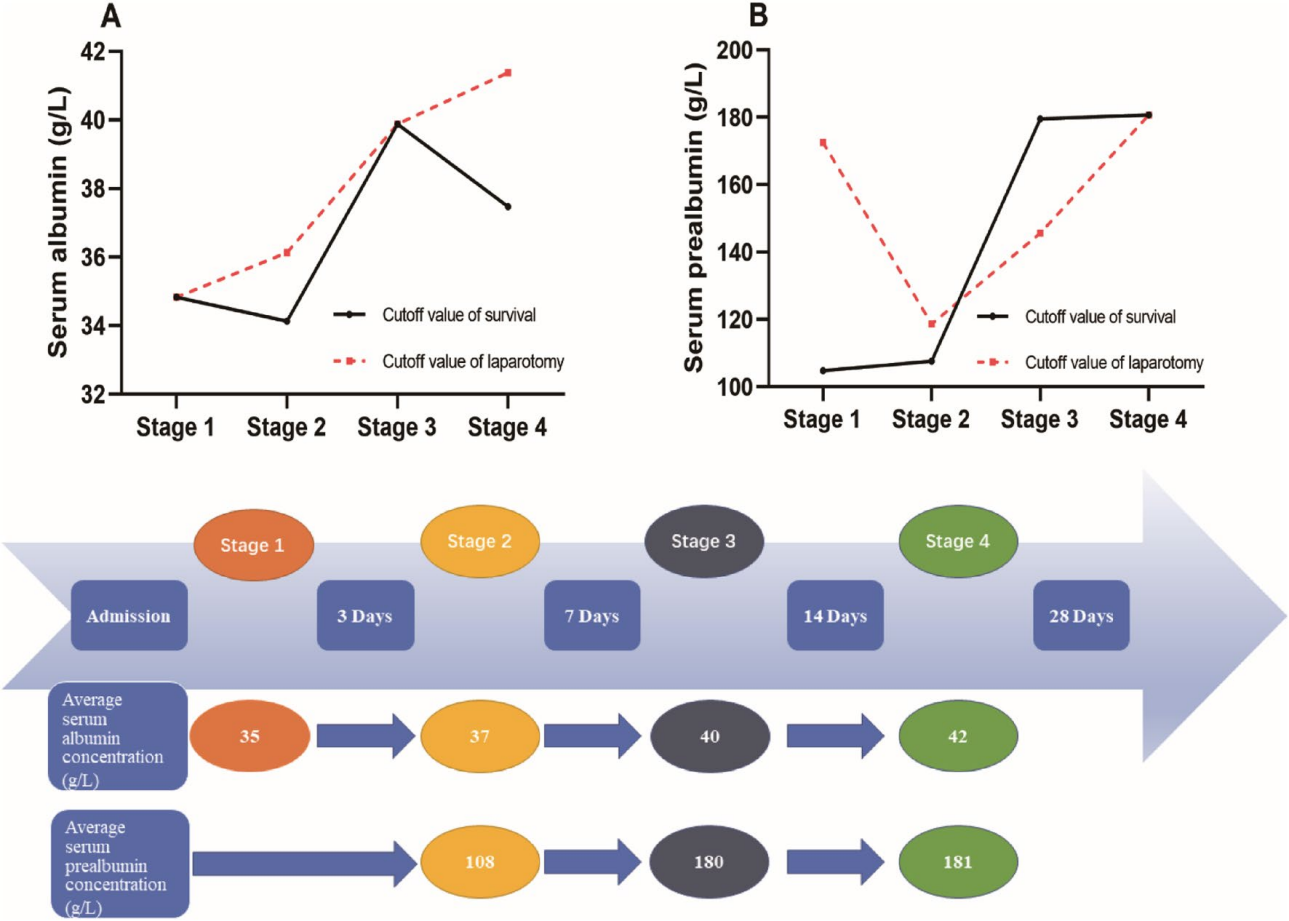
Summary of the albumin and prealbumin cut-off values at different stages of SAP. The upper figure shows the cut-off value tendency of serum albumin/prealbumin. The higher values of each stage are listed in the lower figure as the goal to be reached during clinical treatment.

After the data from the enrolled patients were analyzed by ROC and the cut-off values were calculated, the albumin/prealbumin cut-off values of different stages were summarized, as shown in Fig. 6. The upper parts of Fig. 6 are the curves linked by cut-off values, and the lower part summarizes the result of each defined stage. Except for the prealbumin value at the very first stage, which lacks significant predictive ability, all values can represent a treatment goal in each stage.

According to the results summarized in Fig. 6, the lack of significance of the prealbumin cut-off value in stage 1 can be explained by the fact that in this stage, patients’ basic value varies greatly because of their basic nutrition state and unmeasurable disease consumption. Thus, it fulfils the clinical reality. With the development of disease and the progress of the treatment, the serum albumin should be elevated to at least 35 g/L at 3 days after onset, 37 g/L at 4–7 days, 40 g/L at 8–14 days and 42 g at 15–28 days, and correspondingly, the serum prealbumin level should be greater than 108 g/L at 4–7 days, 180 at 8–14 days and 181 at 15–28 days.

The increasing range of cut-off values from stage 2 to stage 3 was the greatest compared with the other stages. From 7 to 14 days after the onset of SAP, the serum albumin and prealbumin concentrations should be rapidly elevated to maintain a high level in stages 3 and 4, in which the cut-off curves were flattened out. In our recent knowledge from the intensive management of SAP, controlled fluid resuscitation should be undertaken; moreover, to make the patient reach a stable state as soon as possible, the blood volume should be expanded at a crystal-to-colloid ratio of 2:1^3,5^. Because of its potential benefit, exogenous albumin is always the preferred choice over artificial colloids^10^. Based on our study, after the acute phase, 4–7 days after onset is a crucial period of time to fill up supplementary albumin by infusion and elevate the albumin level to over 37 g/ml in time. On the other hand, this suggests that prealbumin is also elevated to over 108 g/L. The recovery of prealbumin relies on the amelioration of the nutritional state. Since enteral nutrition (EN) makes the main contribution to elevating the prealbumin level, we suggest that EN be started at this stage, and it is also one of the theories to support the early beginning of EN for SAP patients^14,15^.

Also, besides the nature colloid infusion in early resuscitation stage, good prognosis of SAP relied on appropriate supportive treatment, especially enteral nutrition. The timing of initiation is that the intra-abdominal pressure is less than 20 mmHg, the intestinal tract has been cleared, 3–5 days after the onset, and no more than 7 days at the latest. All patients were fed by nasojejunal feeding to prevent reflux. 4 patients died early and could not be evaluated. The mean time to initiation of enteral nutrition in all these patients was 3.99 ± 2.08 days after admission. The initiation time of enteral nutrition in survival and non-survival group was 3.91 ± 1.97 and 4.48 ± 2.63 days, with no statistical significance (P = 0.270).

On the other hand, the predictive values of classic scoring systems were also calculated in this study. It disclosed that APACHE II score, BISAP score, and modified Marshall score had larger AUCs in ROC curves which meant higher predicting values in prognosis. But with the development of the disease, the level of albumin/prealbumin showed their advantages in predicting.

In this study, since the effects of cut-off values set as thresholds were validated by Kaplan–Meier survival curves and log-rank tests (Figs. 4 and 5), we propose an albumin goal-directed therapy for SAP patients, limited by the sample scale. A more accurate goal for the albumin and prealbumin concentration depends on a larger scale or prospective study. However, the present study helps us clarify the following unanswered questions. First, although the debate on crystals versus colloids in fluid resuscitation is still ongoing, exogenous supplementation with albumin surely benefits SAP patients. Second, the situation of blind supplementation with albumin can be changed, and a definite goal has herein been set to guide the clinical treatment. Third, one more theory is proposed to provide evidence of the advantage of enteral nutrition in the early stage of SAP. Fourth, after fluid resuscitation at 72 h after onset, albumin is still suggested to be infused continuously until reaching the goal in the different stages, as mentioned above. Fourth, unlike classical scoring system, serum albumin/prealbumin level began to show their predictive value from sub-acute stage of SAP instead of onsetting stage. Finally, as albumin infusion and EN support are routine and easily obtained measures in SAP treatment, the goal is to direct albumin- and prealbumin-elevating treatment, which can improve the prognosis and benefit patients with SAP at a relatively low cost.

## Conclusion

Two main questions in the process of SAP have been answered by our study. First, exogenous supplementary albumin, which acts as colloid material, is an indispensable element in the early stage of fluid resuscitation of SAP and should be continuously provided until the serum concentration reaches 37 g/L 7 days after onset. Furthermore, the albumin level should be kept over 40 g/L in the following stage. Second, from the viewpoint that serum prealbumin should be elevated to 108 g/L in 7 days and 180 g/L in 14 days, early initiation of enteral nutrition undoubtedly benefits SAP patients. However, the concentration values of albumin and prealbumin need to be more accurately adjusted by further research. Maintaining a high level of these two clinical parameters definitely contributes to a better prognosis of SAP.

### Limitations

Because it was a retrospective study, we could not control for the variable of colloid infusion volume. The colloids infused during the treatment included artificial colloids, albumin and plasma, which made it difficult to accurately calculate the infusion volume of albumin. Therefore, we used the results of colloid infusion (serum albumin concentration) as the research object to evaluate the prognosis of SAP patients. Although ICU stay durations were important indicator for judging prognosis, we still did not use it in this article. Since our ICU undertook various stages of treatment after the onset of SAP, including dressing changes after surgery and the recovery stage with stable vital sign because of the short of beds in general wards, which significantly prolonged ICU length of stay and weakened the significance of this indicator. So, we lack precise ICU duration which reflect the real situation of the patients.

As to the data base, we also have to explain the reason for the absence of external validation. Ideally, external validation would typically require the use of 1/3 of the cases in the database, but since we have modeled data from all patients, this is a shortcoming of our study.

## Methods

### Ethics statement

This study was approved by the Ethics Committee of Ruijin Hospital, Shanghai Jiao Tong University School of Medicine and granted waiver of informed consent (KY2021-325). Data analysis was performed in accordance with the principles expressed in the 1964 Helsinki Declaration and its later amendments.

### Study design and patient enrolment

**Figure 7:**
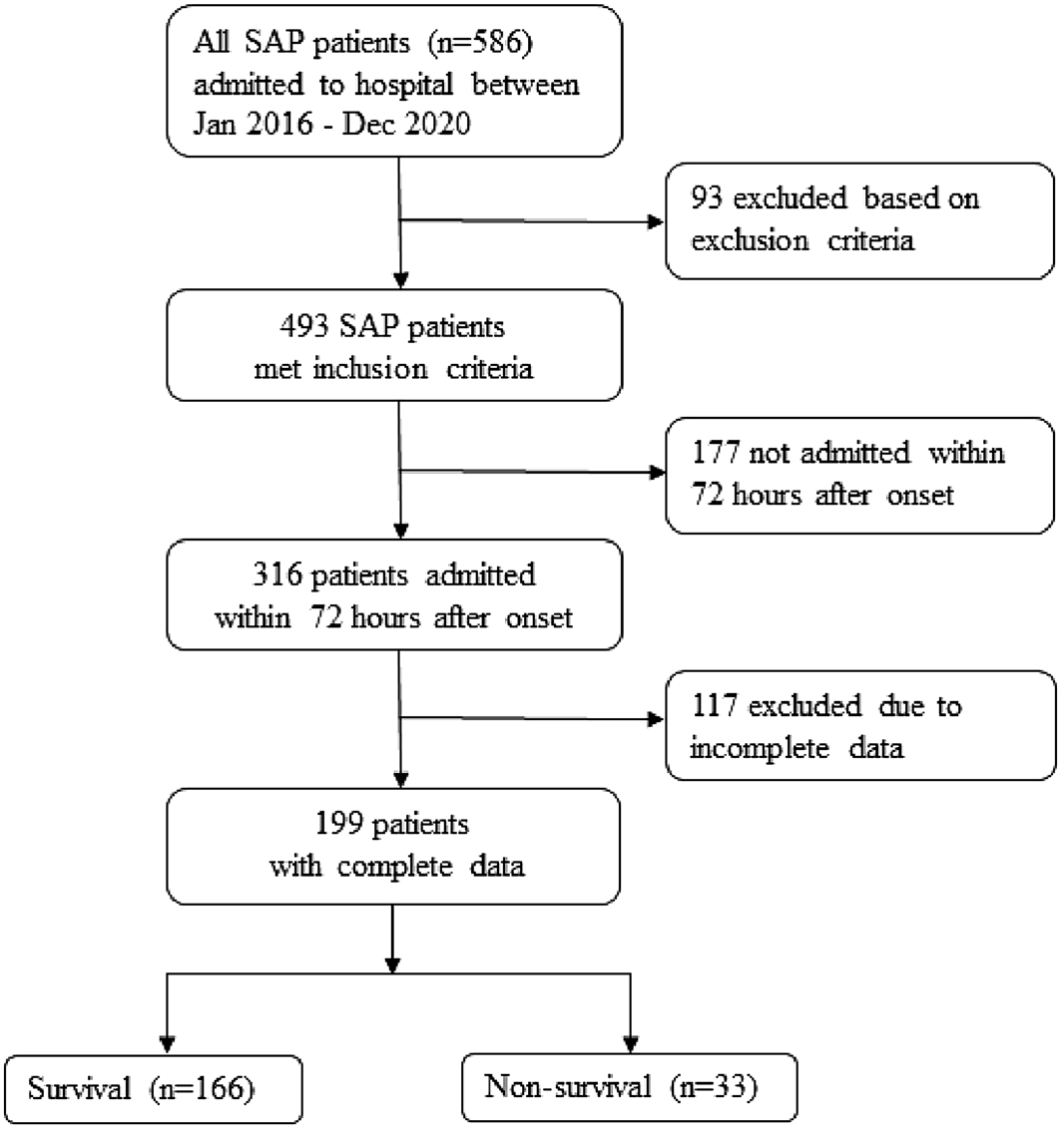
Procedures for patient enrolment and screening.

All 586 SAP patients admitted to our hospital between Jan 2016 and Dec 2020 were screened by the previously designed process of enrolment (Fig. 7). The inclusion criteria included two aspects. First, all SAP patients fulfilled the diagnosis and classification mentioned in 2013 Guidelines of the International Association of Pancreatology. Thus, all patients had two or more manifestations as follows: clinical symptoms (epigastric pain); laboratory results (the level of serum amylase or lipase was more than three times the upper limit of the normal value); and/or imaging observations (CT, MRI, ultrasonography). Meanwhile, all the patients had experienced persistent organ failure lasting more than 48 h. Second, all enrolled patients had to have been admitted to our hospital within 72 h after the onset of pancreatitis. In order to avoid the bias, patients first visiting to our hospital or without previous SAP targeted treatment were preferred. The exclusion criteria were patients with complications, including pregnancy; tumors; chronic pancreatitis; and basic disease causing severe cardiac, kidney and liver dysfunction. Of 586 patients, 93 were excluded by the exclusion criteria, 177 were excluded because of late admissions more than 72 h after onset, and 117 were excluded due to incomplete clinical data. A total of 199 patients fulfilled the criteria and were enrolled. All enrolled patients fulfill the SAP criteria and with newly onsetting organ dysfunctions caused by SAP rather than chronic basic disease. Figure 7 shows the patient enrolment process.

This study aims to discuss the relationship between the SAP patients’ level of serum albumin/prealbumin and prognosis, including the rate of nonlaparotomy and survival, and whether the intentional elevation of albumin/prealbumin concentrations can decrease the laparotomy rate and mortality. Furthermore, we tried to set the goal of serum albumin/prealbumin, which may influence the prognosis at different stages and achieve more targeted colloid infusion treatment. In this study, the course of the disease was divided into 4 stages along the timeline after the onset of SAP. The first stage was defined as 0 days to 3 days after onset. The second stage was 4 days to 7 days. The third stage was 8 days to 14 days, and the fourth stage was 15 days to 28 days. In every stage, according to the different situations, the times of serum albumin/prealbumin examination varied in different patients; consequently, the average serum albumin/prealbumin concentration in each stage was calculated, representing the overall albumin/prealbumin level in the corresponding stage of the patients.

### Statistical analysis

At the beginning of this study, the descriptive data were calculated according to survival or nonsurvival after in-hospital treatment. A total of 199 patients were divided into a survival group and a nonsurvival group. A series of descriptive data were recorded, including basic information, SAP aetiology, appearance of comorbidities, laboratory exam indicators and clinical evaluation scores, especially for SAP. To determine the difference between variables in the two groups, Pearson Chi-squared and Mann–Whitney U tests were used. Moreover, to determine the relationship between serum albumin/prealbumin levels and prognosis, receiver operating characteristic (ROC) curves were drawn. AUCs were calculated to reflect the relationship between the average albumin/prealbumin level and prognosis, including nonlaparotomy and survival 28 days after SAP onset. Meanwhile, the cut-off values for different stages were considered to be the threshold or treatment goal, and the prognosis were expected to be ameliorated if the goals were reached or exceeded^16,17^. On the other hand, ROC curves and AUCs were also done which reflecting the relationship between traditional scoring system (APACHE II score, BISAP score, and modified Marshall score) laparotomy and survival results. Finally, Patients were divided into on-target and non-target groups according to whether reach the albumin or prealbumin cut-off value. Kaplan–Meier survival curves and log-rank tests were used to compare mortality between two groups. We performed all statistical tests using SPSS 19 (IBM, Armonk, NY, USA) software and considered P values of < 0.05 to be statistically significant for all results.

### Standardized early-stage fluid resuscitation

All SAP patients experienced standardized resuscitation therapy on admission according to the guideline of our center. Fluid resuscitation was divided into two phases, the volume expansion phase and the fluid distribution adjustment phase. The volume expansion phase was generally completed within 24 h of admission to the ICU. Two vascular accesses were established and fluid infusion was performed simultaneously. If MAP was < 60 mmHg, vasoactive drugs were used to increase it to > 60 mmHg and the infusion rate was increased to 5–10 mL/kg/h. Blood volume expansion was considered to reach the standard if two or more of the following requirements were met: heart rate < 120 bpm; MAP 65 to 85 mmHg; urine output ≥ 0.5 ml/kg/h; or HCT 30% to 35%. Assessments were required every 4–6 h to avoid overhydration. After the blood volume expansion reached the required standard, the body fluid distribution was adjusted rapidly. The infusion ratio of crystalloid to colloid was 2–4:1 based on the physiologically required amount of fluid. Diuretics or renal replacement therapy were used to remove the excess fluid injected during the volume expansion phase from the body. The endpoint of fluid resuscitation was defined as the disappearance of the SIRS response. All albumin doses were decided by clinical requirements and the study did not involve any adjustment or difference in clinical treatment.

## Data Availability

The data that support the findings of this study are available from the corresponding author upon reasonable request in a de-identified manner.
